# Exploring the potential central regulatory mechanisms of acupuncture for acute-stage Bell’s palsy: an fMRI-based investigation

**DOI:** 10.3389/fnins.2025.1647538

**Published:** 2025-09-24

**Authors:** Xiao-shuang Xu, Ya-ting Zhang, Xiao-wei Li, Yu-ling Shu, Jing-can Zhang, Ting-ting Miao, Yan-yan Yang, Jun Yang, Hai-ping Shi

**Affiliations:** ^1^Anhui University of Traditional Chinese Medicine First Clinical Medical College, Hefei, China; ^2^Department of Imaging, The First Affiliated Hospital of Anhui University of Traditional Chinese Medicine, Hefei, China; ^3^Department of Acupuncture and Rehabilitation, The First Affiliated Hospital of Anhui University of Traditional Chinese Medicine, Hefei, China; ^4^Department of Tuina, The First Affiliated Hospital of Anhui University of Traditional Chinese Medicine, Hefei, China

**Keywords:** Bell’s palsy, acupuncture, functional magnetic resonance imaging (fMRI), fractional amplitude of low-frequency fluctuation (fALFF), regional homogeneity (ReHo)

## Abstract

**Objective:**

This study utilized resting-state functional magnetic resonance imaging (fMRI) to examine changes in brain functional activity following acupuncture treatment for acute Bell’s palsy (BP) and to investigate the potential central regulatory mechanisms involved.

**Methods:**

A total of 55 patients with acute Bell’s facial paralysis (within 1–7 days of onset) were enrolled in the patient group, while 48 individuals without the condition were included as the healthy control group. The patient group received acupuncture therapy at EX-HN16 (*Qianzheng)*, SJ17 (*Yifeng*), ST2 (*Sibai*), GB14 (*Yangbai*), EX-HN4 (*Yuyao*), SI18 (*Quanliao*), ST6 (*Jiache*), ST4 (*Dicang*), ST8 (*Touwei*), and bilateral LI4 (*Hegu*) points on the affected side. Each session lasted 30 min and was administered three times a week (Wednesday, Friday, and Sunday) until day 28 of the disease course. The patient group underwent fMRI scans, House–Brackmann (H-B) grading, Sunnybrook scale evaluation, and facial disability index (FDI) assessment both prior to the initial treatment and on the 28th day. The healthy group received a single fMRI scan after enrollment. MATLAB R2017 software was used to calculate the fractional amplitude of low-frequency fluctuation (fALFF) and regional homogeneity (ReHo) in patients before and after treatment, as well as in healthy controls.

**Results:**

Following treatment, the patient group showed significant improvements in H-B, Sunnybrook, and FDI scores compared to pretreatment levels (*P* < 0.05), with an overall effective rate of 96.4% (53/55). Prior to treatment, compared to healthy controls, patients exhibited decreased fALFF in the right posterior cingulate gyrus, increased fALFF in the right postcentral gyrus, left and right middle frontal gyri, and increased ReHo in the left precentral gyrus, right postcentral gyrus, and left middle occipital gyrus. After treatment, when compared to healthy controls, patients showed decreased fALFF in the left and right medial superior frontal gyri, and increased fALFF in the right postcentral gyrus, left precentral gyrus, and bilateral lingual gyri, and increased ReHo in the right precentral gyrus, bilateral transverse temporal gyri, right lingual gyrus, and right thalamus, and decreased ReHo in the right middle frontal gyrus. Relative to pretreatment values, patients displayed decreased fALFF in the left medial superior frontal gyrus and increased fALFF in the left precentral gyrus. Additionally, ReHo decreased the right and left medial superior frontal gyri, while it increased in the right inferior parietal angular gyrus, right precentral gyrus, and left superior parietal gyrus.

**Conclusion:**

Acupuncture demonstrates a clear therapeutic effect on acute BP and contribute to clinical symptom improvement. Marked differences in brain functional activity were observed between patients and healthy individuals. The therapeutic effect of acupuncture may be linked to its ability to facilitate functional reorganization in brain regions associated with sensation, movement, and emotion.

**Clinical trial registration:**

https://www.chictr.org.cn/searchproj.html? title=&officialname=&subjectid=&regstatus=&regno=ChiCTR2200065223& secondaryid=&applier=&studyleader=&createyear=&sponsor=&secsponsor= &sourceofspends=&studyailment=&studyailmentcode=&studytype=&study stage=&studydesign=&recruitmentstatus=&gender=&agreetosign=&measure= &country=&province=&city=&institution=&institutionlevel=&intercode=& ethicalcommitteesanction=&whetherpublic=&minstudyexecutetime=&maxstudy executetime=&btngo=btn, identifier ChiCTR2200065223.

## 1 Introduction

Bell’s palsy (BP) is an acute idiopathic peripheral facial paralysis characterized by a sudden onset of weakness or paralysis on one side of the face. It is among the most frequently encountered facial nerve disorders in clinical settings, accounting for approximately 60%–75% of all facial paralysis cases (Peitersen, 2002). Although its exact cause remains unclear, it is generally thought to be associated with inflammation, edema, and compression of the facial nerve triggered by viral infection (Mu et al., 2022), such as the reactivation of herpes simplex virus type 1. Regarding treatment (Gagyor et al., 2019), glucocorticoids are considered the first-line therapy due to their effectiveness in reducing nerve swelling, and their combination with antiviral medications may further enhance patient outcomes. Additionally, physical therapy and surgical decompression should be tailored based on individual patient conditions. Acupuncture therapy (An et al., 2025) for BP has been shown to support the recovery of facial nerve function, alleviate facial symptoms, improve therapeutic outcomes, and has relatively few adverse effects. Nonetheless, the underlying mechanisms by which acupuncture exerts these effects remain unclear (Ma et al., 2021), necessitating further investigation.

Magnetic resonance imaging (MRI) serves as one of the objective indicators for evaluating BP (Wang Y. et al., 2021). Functional MRI (fMRI) is a key technique for investigating brain activity in humans (Glover, 2011). In recent years, fMRI has proven particularly valuable for studying the dynamic interactions within brain networks, neural plasticity, and mechanisms underlying traditional medical treatments (Li and Liu, 2010). It is a useful tool for exploring the central mechanisms of acupuncture. Moreover, the effects of acupuncture are closely linked to the central nervous system (Cai et al., 2018; You et al., 2013). Some researchers have utilized fMRI to investigate the mechanism of BP and have suggested that it can dynamically monitor central compensatory processes (Calistri et al., 2021). Fractional amplitude of low-frequency fluctuation (fALFF) and regional homogeneity (ReHo) are analytical methods based on resting-state fMRI used to assess localized brain function. Together, these methods offer a more comprehensive evaluation of functional alterations in specific brain areas. Therefore, building on the assessment of acupuncture’s clinical efficacy in treating BP, this study employed fALFF and ReHo to examine central nervous system dysfunction and its underlying mechanisms in BP, aiming to investigate how acupuncture influences brain function in BP patients and to provide an objective foundation for its clinical application.

## 2 Materials and methods

### 2.1 Research design

This study was conducted as an experimental intervention. Individuals with acute BP, recruited from the general population, were included in the BP group (BP patients). Concurrently, healthy controls (HCs) were recruited, matched to the BP group in terms of age, sex, and handedness. None of the HC participants had immediate family members with a history of BP.

All participants underwent an initial fMRI examination with typical findings and provided written informed consent in accordance with the guidelines set by the Ethics Committee of the First Affiliated Hospital of Anhui University of Traditional Chinese Medicine (Ethical Approval No.: 2022AH-22). The clinical trial was registered under ChiCTR2200065223. Participants were enrolled only after registration, ethics approval, and a thorough explanation of the study protocol. Personal information was kept confidential, and participants retained the right to withdraw from the study at any point. Because of the nature of acupuncture treatment, blinding of acupuncturists was not feasible. However, outcome assessors were blinded to group allocation and treatment status. They received standardized training to ensure scoring consistency and were instructed not to ask patients about treatment details. Additionally, the fMRI data analysts were blinded to group allocation and time points during preprocessing and statistical analysis.

### 2.2 Inclusion and exclusion criteria

#### 2.2.1 Inclusion criteria

1. Acute BP patients: Diagnosed with Bell’s facial paralysis based on established diagnostic criteria (Jia, 2013); first-time unilateral BP with an H-B facial nerve grade higher than I but lower than VI, with symptom onset within the past 7 days; no acupuncture treatment received in the past 3 months; aged between 16 and 65 years, of any sex, right-handed; years of education ≥12 years; routine MRI scans show no significant intracranial abnormalities; eligible for MRI scanning (i.e., no metal dental work, pacemakers, stents, or claustrophobia); normal mental and behavioral status, without major systemic diseases; and provided voluntary informed consent and were able to comply with and complete the acupuncture treatment.

2. Healthy subjects: Healthy individuals matched in age, sex, years of education, and other characteristics with the BP patients, right-handed; no acupuncture treatment within the past 3 months; normal neurological examination findings, no evident abnormalities on routine MRI scans, and no personal or family history of neurological or psychiatric disorders; eligible for MRI scanning (i.e., no metal dental work, pacemakers, stents, or claustrophobia); and voluntarily signed informed consent.

#### 2.2.2 Exclusion criteria:

1. Acute BP patients: Individuals with facial paralysis secondary to other conditions (e.g., tumors, trauma, or otitis media); those with comorbid underlying diseases, severe organic disorders of major organs, or mental disorders; pregnant or breastfeeding women; and those with poor compliance or inability to complete the study.

2. Healthy subjects: Individuals with underlying diseases, severe organic disorders of major organs, or mental disorders; pregnant or lactating women; those with a history of facial trauma, otitis media, or viral infection (e.g., herpes simplex) within the past 3 months; those with a family history of neurological diseases (not limited to BP); and individuals currently taking medications that affect central nervous system function (e.g., antidepressants, corticosteroids).

### 2.3 Clinical evaluation scale

#### 2.3.1 H-B scale

Grade I: normal function

Grade II: mild dysfunction

Grade III: moderate dysfunction

Grade IV: moderately severe dysfunction

Grade V: severe dysfunction

Grade VI: complete paralysis (House and Brackmann, 1985)

#### 2.3.2 Sunnybrook facial nerve rating scale

The static assessment evaluates three regions: the eye, cheek, and mouth. Scoring ranges are as follows: eye 0–3 points, cheek 0–4 points, and mouth 0–2 points. The static score is calculated as total score × 5.

Dynamic assessment includes five facial movements: raising the forehead, gently closing the eyes, smiling with an open mouth, wrinkling the nose, and puckering the lips. This part evaluates both voluntary movement and synkinesis. Voluntary movement is scored from 1 to 5 points per movement, with the voluntary movement score computed as total score × 4. Synkinesis is graded from 0 to 3 points, with the synkinesis score being the total of these values. The dynamic assessment score is calculated as voluntary movement score–static score–synkinesis score. The total possible score ranges from 0 to 100, with higher scores indicating better facial nerve function (Li, 2005).

#### 2.3.3 Facial disability index (FDI) score

This index includes two components: physical function (FDIP) and social/well-being function (FDIS). FDIP has a total score ranging from 0 to 25, where higher scores reflect better physical motor function. FDIS scores range from 5 to 30 points, with lower scores indicating better social function and quality of life (Deng et al., 2017).

### 2.4 Evaluation criteria of curative effect

Based on the H-B scale (House and Brackmann, 1985), treatment outcomes were categorized as follows: *Cured*: H-B grade I; *Markedly effective*: H-B grade II; *Effective*: H-B grade III; *Ineffective*: H-B grades IV–VI.

### 2.5 Acupuncture intervention measures

Patients in the BP group received standard acupuncture treatment.

**FIGURE 1:**
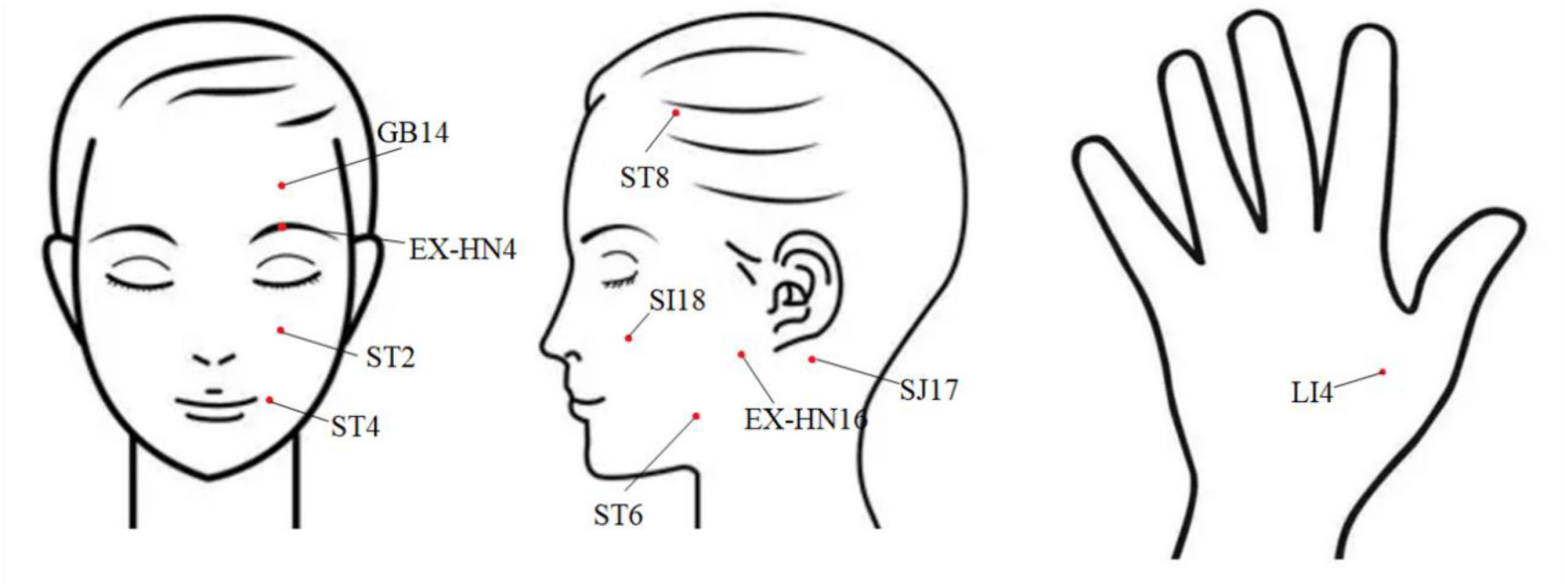
Distribution of acupoints on the face and hands.

Acupoint selection: In accordance with the guidelines outlined in *Acupuncture* (Liang and Wang, 2016), the selected acupoints on the affected side included ST2 (*Sibai*), GB14 (*Yangbai*), ST8 (*Touwei*), EX-HN16 (*Qianzheng*), SJ17 (*Yifeng*), SI18 (*Quanliao*), ST6 (*Jiache*), ST4 (*Dicang*), EX-HN4 (Yuyao) and bilateral LI4 (*Hegu*) (Figure 1).

Procedure: After disinfecting the local skin, patients were placed in a supine position. Disposable acupuncture needles (0.25 mm × 25–40 mm; Wujiang Yunlong Medical Equipment Co., Ltd., Su Jizhun 20142200226) were used. ST2: inserted perpendicularly 10–15 mm. GB14: inserted 20–25 mm toward the EX-HN4 region. ST8: inserted horizontally 10–15 mm in an upward direction. SI18: inserted obliquely 10–15 mm toward the anterior aspect of the lower root of the ear on the affected side. SJ17: inserted perpendicularly 12–20 mm. SI18: inserted perpendicularly 12–20 mm. ST6 and ST4: inserted bidirectionally 15–20 mm. LI4: inserted perpendicularly 12–20 mm.

Criteria for assessing “*Deqi*” (Liang and Wang, 2016): “*Deqi*” was defined as the simultaneous occurrence of two conditions: (1) needle sensations reported by the patient (e.g., soreness, numbness, distension, heaviness) and (2) a tight, rough sensation under the needle perceived by the acupuncturist (i.e., “needle sensation”). Once the arrival of qi was achieved, the needles were retained for 30 min. Treatment sessions were conducted three times per week (Wednesday, Friday, and Sunday) and continued until the 28th day from the onset of symptoms. If a patient was cured within the 28-day period, treatment was concluded early; if not, treatment continued beyond that point. All acupuncture procedures were carried out by the same practitioner, who had more than 3 years of clinical experience.

### 2.6 fMRI data acquisition

All fMRI data in this study were collected using an 8-channel head coil on a 3.0T Discovery MR750 scanner (GE, USA) at the Imaging Center of the First Affiliated Hospital of Anhui University of Traditional Chinese Medicine. These scans were performed by a trained technician. The BP group underwent two fMRI scans: one before the first acupuncture treatment and another on the 28th day of the disease course. The HC group underwent a single fMRI scan after enrollment. Before scanning, participants removed all metal and magnetic objects in the preparation room. After sufficient relaxation, they entered the scanning room in a calm state and wore earplugs to reduce noise. Subjects were instructed to lie flat with their eyes closed; keep their entire body, especially the head, still; remain awake without engaging in any active thinking; and breathe evenly. The fMRI procedure included both structural and functional imaging.

Structural image parameters: Structural images were obtained using a 3D T1 BRAVO sequence with the following parameters: repetition time (TR) = 8.2 ms, echo time (TE) = 3.2 ms, inversion time = 450 ms, flip angle = 12°, scanning field = 256 mm × 256 mm, matrix size = 256 × 256, slice thickness = 1.0 mm, slice gap = 1.0 mm, total of 200 slices acquired in the sagittal plane. The total scan duration was 5 min and 36 s.

Functional imaging parameters: Functional images were acquired using a gradient echo planar imaging sequence with TR = 2000 ms, TE = 30 ms, flip angle = 90°, scanning field = 224 mm × 224 mm, matrix size = 64 × 64, slice thickness = 3.0 mm, voxel size = 3 mm × 3 mm × 3 mm, total of 36 slices obtained axially. The total acquisition time was 6 min and 10 s.

### 2.7 fMRI data preprocessing

Imaging data in this study were processed using dcm2niigui and the DPABI software package within MATLAB R2017b. First, the data in DICOM format were converted to NIFTI format. The “co-” data (with the neck removed) were selected, and each dataset was checked for artifacts or excessive head movement. Next, resting-state fMRI data were preprocessed using DPABI software with the following steps: (1) Time correction by discarding the first 10 time points. (2) Head motion correction: Head motion was quantified using framewise displacement (FD). Time points with FD > 0.5 mm were excluded, and participants with >20% of time points discarded were removed from further analysis. Both mean FD and maximum rotation angle were calculated, and datasets with head motion exceeding 3 mm or 3° were excluded. (3) Spatial normalization by registering data to the Montreal Institute of Neuroscience template and resampling to a voxel size of 3 mm × 3 mm × 3 mm. (4) Regression of nuisance signals, including head motion parameters, white matter, and cerebrospinal fluid signals. (5) Spatial smoothing using a Gaussian kernel with a full width at half maximum of 6 mm × 6 mm × 6 mm. (6) Temporal filtering to retain low-frequency signals with the range 0.01–0.08 Hz.

### 2.8 Statistical analysis

SPSS 25.0 software was used to analyze the general data and clinical scale data of the BP and HC groups. Measurement data were tested for normality and homogeneity of variance. If both assumptions were met, independent sample *t*-tests and paired *t*-tests were applied. If these conditions were not met, the Wilcoxon rank sum test was used. Categorical data were analyzed using the chi-squared test. A *P*-value of < 0.05 was considered statistically significant. DPABI software was utilized to conduct two-sample independent *t*-tests comparing brain imaging indicators between BP patients before and after acupuncture and HCs. Paired *t*-tests were conducted to compare brain imaging indicators before and after acupuncture treatment in patients. The final results were corrected using Gaussian random field (GRF) theory with thresholds set at voxel-level *P* < 0.001 and cluster-level *P* < 0.01.

## 3 Results

### 3.1 Demographic characteristics of participants

Between September 2023 and September 2024, a total of 103 participants were recruited from the First Affiliated Hospital of Anhui University of Traditional Chinese Medicine, including 55 patients in the BP group and 48 in the HC group. None of the participants dropped out. All subjects completed the fMRI scans, and all 55 patients underwent acupuncture treatment without any serious adverse effects. There were no significant differences in age, sex, or years of education between the two groups. The demographic details of all participants are presented in Table 1.

**TABLE 1:** Comparison of general data between the BP and HC.

Group	n	Sex/n		Age/years	Years of education
		Male	Female	Average ($\bar{\text{x}}\pm s$)	Average ($\bar{x}\pm \text{s}$)
BP	55	32	23	37.38 ± 10.35	13.89 ± 2.57
HC	48	23	25	37.65 ± 11.14	13.63 ± 2.13
*t/x* ^2^ */Z*		1.085		−0.056	−0.443
*P*		0.298		0.963	0.658

BP, Bell’s palsy; HC, healthy control. Compared with BP and HC, *P* > 0.05.

### 3.2 Comparison of H-B scale, Sunnybrook, and FDI (including FDIP and FDIS) scores before and after treatment in the BP group

Following treatment, the H-B scale score in the BP group showed significant improvement compared to before treatment (*P* < 0.05), as presented in Table 2. Additionally, the Sunnybrook and FDIP scores of patients in the acute phase of BP were higher than those before treatment (*P* < 0.05), while the FDIS scores were lower than before treatment (*P* < 0.05), as shown in Table 3.

**TABLE 2:** Comparison of H-B scale grading before and after treatment in the BP group.

Time	n	H-B					
		Grade I	Grade II	Grade III	Grade IV	Grade V	Grade VI
Before treatment	55	0	4	22	24	5	0
After treatment	55	27	23	3	2	0	0

BP, Bell’s palsy; H-B, House–Brackmann. Compared with before treatment, *P* < 0.05.

**TABLE 3:** Comparison of Sunnybrook, FDIP, and FDIS scores before and after treatment in the BP group.

Time	n	Sunnybrook	FDIP	FDIS
Before treatment	55	38.73 ± 10.35	19.47 ± 2.31	10.38 ± 1.45
After treatment	55	83.22 ± 13.55^1^	24.29 ± 1.15^1^	9.82 ± 0.43^1^
*t/Z*		−24.112	−16.804	−2.566
*P*		0.000	0.000	0.010

FDIP, facial disability index-physical function; FDIS, facial disability index-social/well-being function. Compared with before treatment,

^1^*P* < 0.05.

### 3.3 Clinical efficacy of BP group

Among the 55 patients with acute BP treated, 27 were cured, 23 showed marked improvement, 3 were effective, and 2 were ineffective, resulting in a total effective rate of 96.4%.

### 3.4 fMRI results

**FIGURE 2:**
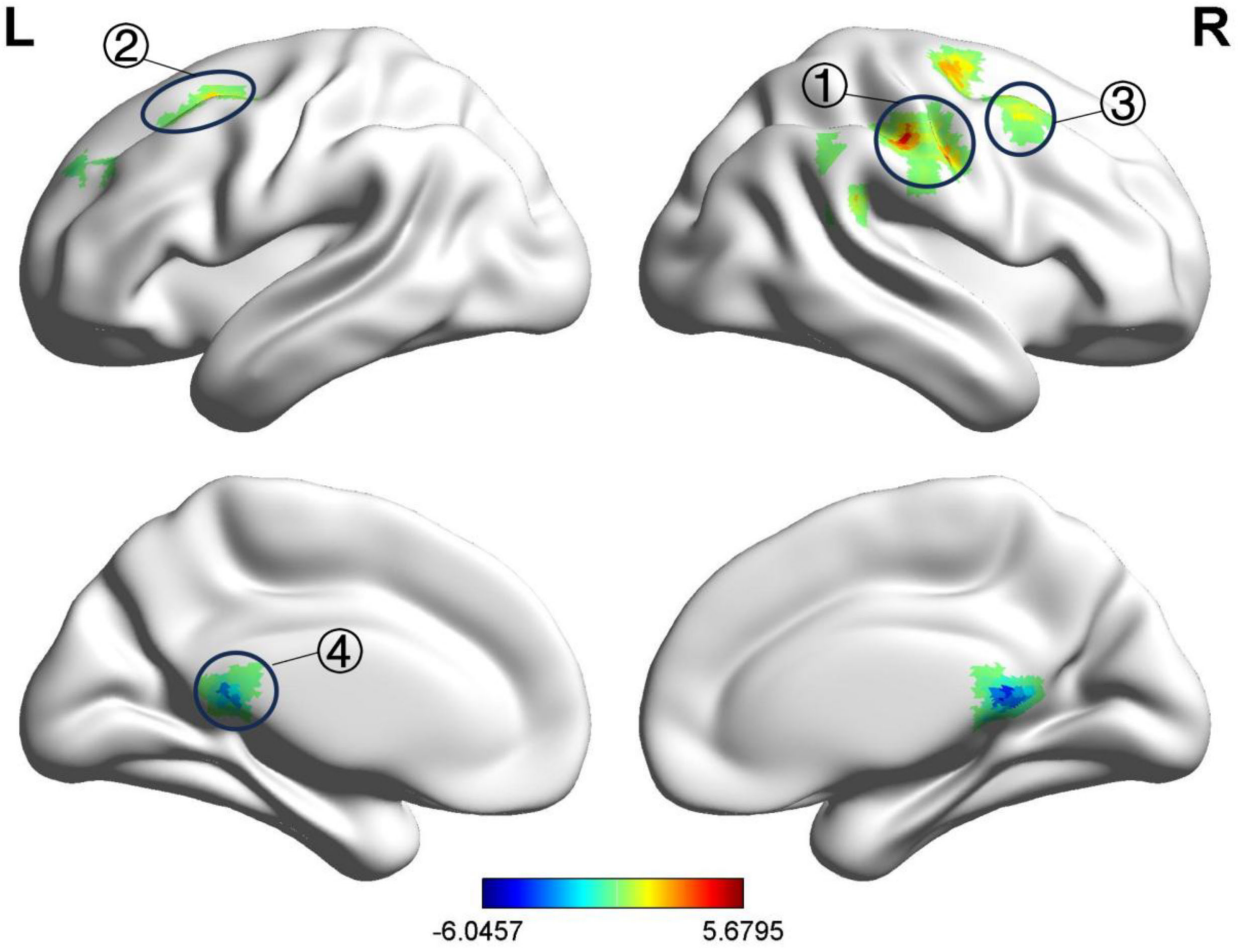
Significant differences in fALFF between before acupuncture treatment BP patients and HC. The result was corrected for GRF with voxel *P* < 0.001, cluster *P* < 0.05. ① Right postcentral gyrus, ② Left middle frontal gyrus, ③ Right middle frontal gyrus Left middle frontal gyrus, ④ Right posterior cingulate gyrus.

**FIGURE 3:**
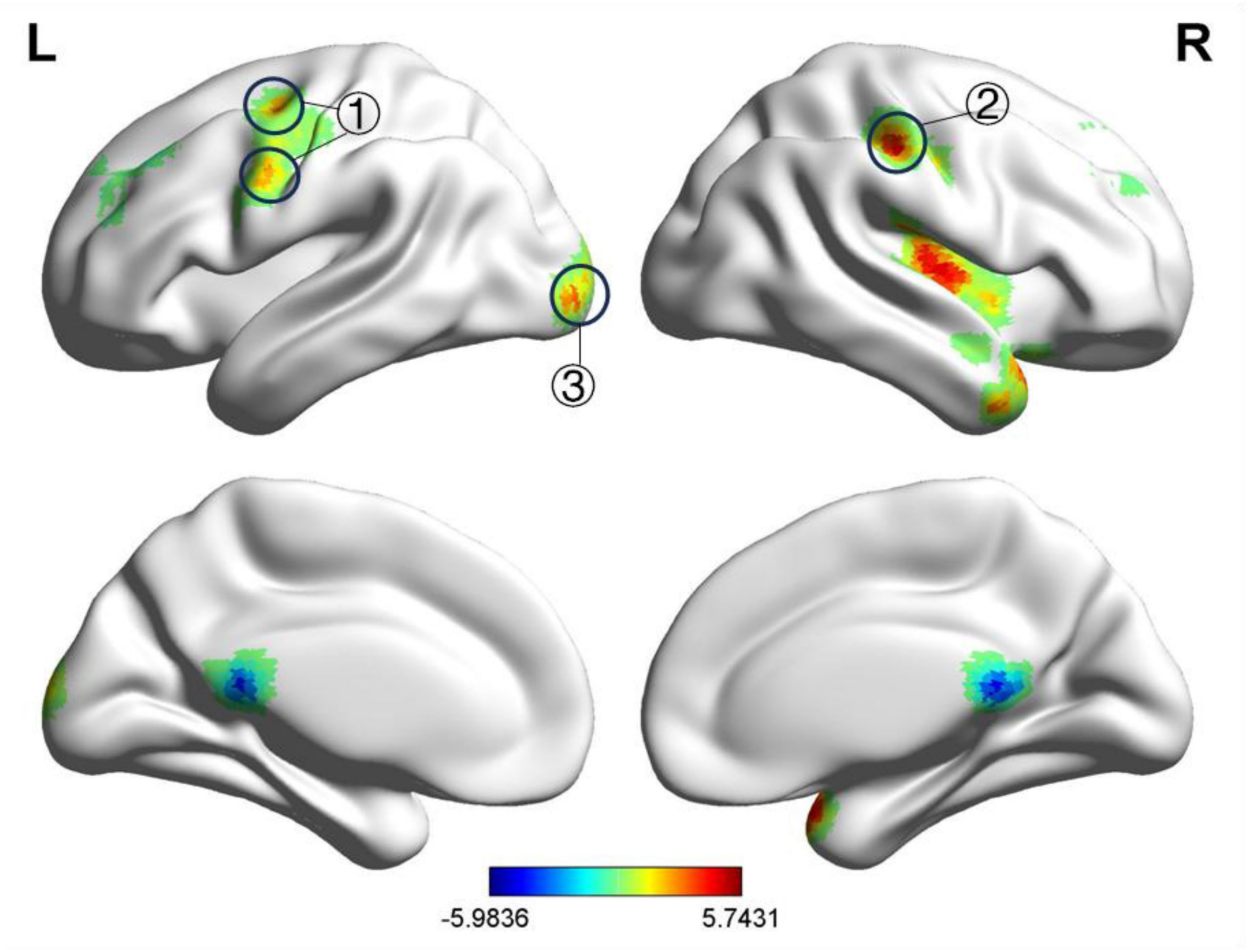
Significant differences in ReHo between before acupuncture treatment BP patients and HC. The result was corrected for GRF with voxel *P* < 0.001, cluster *P* < 0.05. ① Left precentral gyrus, ② Right postcentral gyrus, ③ Left middle occipital gyrus.

Before treatment, compared to the HC group, the BP group showed a decreased fALFF value in the right posterior cingulate gyrus and increased fALFF value in the right postcentral gyrus, left middle frontal gyrus, and right middle frontal gyrus, as detailed in Table 4 and Figure 2. The ReHo values increased in the left precentral gyrus, right postcentral gyrus, and left middle occipital gyrus, as shown in Table 5 and Figure 3. These results were corrected using GRF, with voxel-level *P* < 0.001 and cluster-level *P* < 0.01.

**TABLE 4:** Significant differences in fALFF between before acupuncture treatment BP patients and HC.

Brain Regions	MNI coordinate			Voxel	*t*
	X	Y	Z		
Right postcentral gyrus	51	–24	42	71	5.679
Left middle frontal gyrus	–27	9	51	46	4.981
Right middle frontal gyrus	36	12	48	30	5.319
Right posterior cingulate gyrus	3	–43	8	54	–3.205

BP, Bell’s palsy; fALFF, fractional amplitude of low-frequency fluctuation; HC, healthy control; MNI, Montreal Institute of Neuroscience.

**TABLE 5:** Significant differences in ReHo between before acupuncture treatment BP patients and HC.

Brain regions	MNI coordinate			Voxel	*t*
	X	Y	Z		
Left precentral gyrus	–51	–3	33	98	4.245
Right postcentral gyrus	48	–24	42	69	5.367
Left middle occipital gyrus	–33	–99	0	101	5.004

BP, Bell’s palsy; HC, healthy control; MNI, Montreal Institute of Neuroscience; ReHo, regional homogeneity.

**FIGURE 4:**
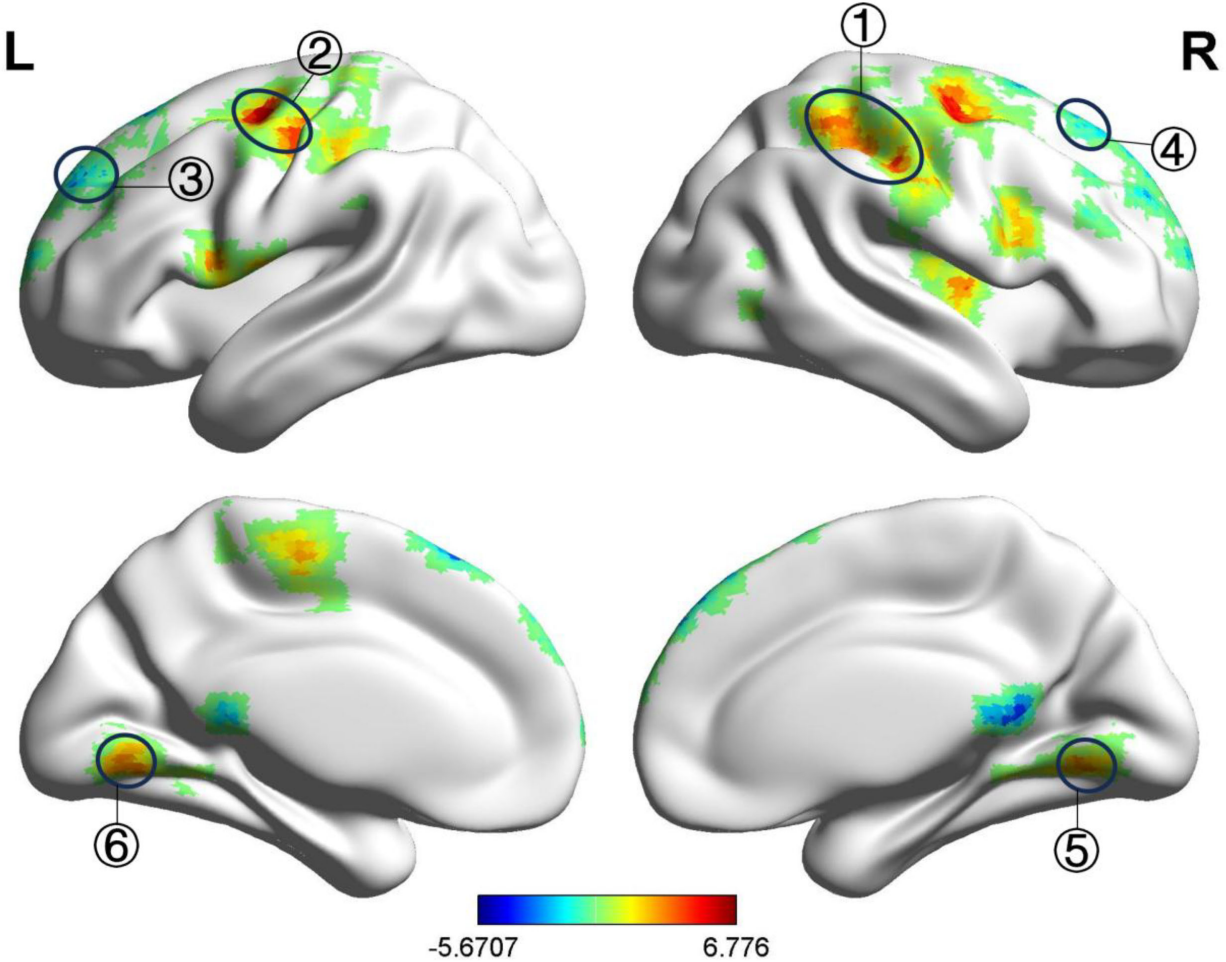
Significant differences in fALFF between after acupuncture treatment BP patients and HC. The result was corrected for GRF with voxel *P* < 0.001, cluster *P* < 0.05. ① Right postcentral gyrus, ② Left precentral gyrus, ③ Left medial superior frontal gyrus, ④ Right medial superior frontal gyrus, ⑤ Right lingual gyrus, ⑥ Left lingual gyrus.

**FIGURE 5:**
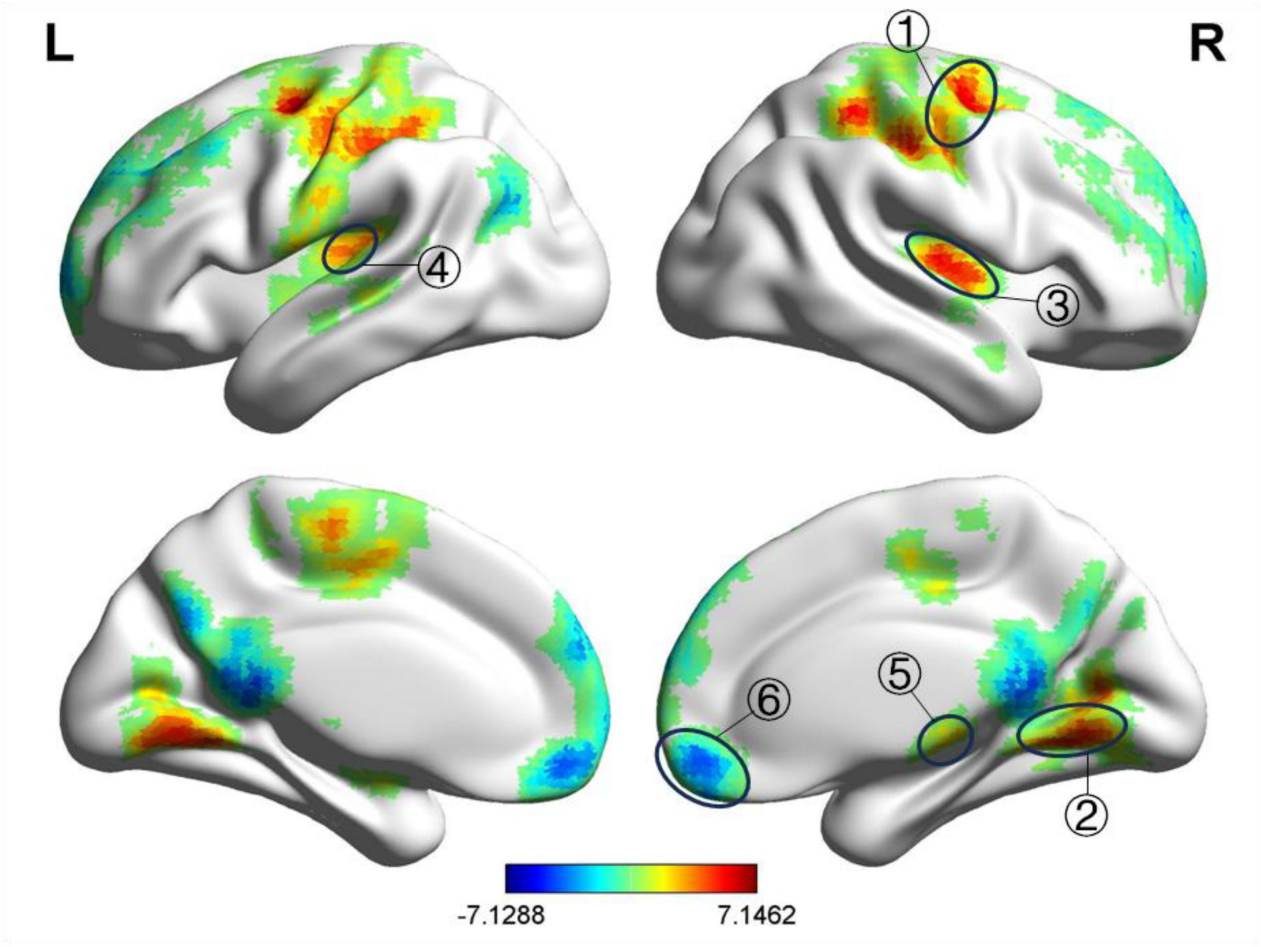
Significant differences in ReHo between after acupuncture treatment BP patients and HC. The result was corrected for GRF with voxel *P* < 0.001, cluster *P* < 0.05. ① Right precentral gyrus, ② Right lingual gyrus, ③ Right transverse temporal gyrus, ④ Left transverse temporal gyrus, ⑤ Right thalamus, ⑥ Right middle frontal gyrus.

After treatment, compared to the HC group, the BP group exhibited decreased fALFF values in the left and right medial superior frontal gyri and increased fALFF values in the right postcentral gyrus, left precentral gyrus, right lingual gyrus, and left lingual gyrus, as shown in Table 6 and Figure 4. The ReHo values were increased in the right precentral gyrus, right lingual gyrus, right transverse temporal gyrus, left transverse temporal gyrus, and right thalamus, as presented in Table 7 and Figure 5. GRF correction was applied with voxel-level *P* < 0.001 and cluster-level *P* < 0.01.

**TABLE 6:** Significant differences in fALFF between after acupuncture treatment BP patients and HC.

Brain regions	MNI coordinate			Voxel	*t*
	X	Y	Z		
Right postcentral gyrus	51	–24	39	476	6.335
Left precentral gyrus	–27	–6	54	353	6.776
Left medial superior frontal gyrus	–3	54	36	174	–3.393
Right medial superior frontal gyrus	27	63	15	116	–3.391
Right lingual gyrus	18	–78	0	72	4.890
Left lingual gyrus	–12	–72	–3	61	5.031

BP, Bell’s palsy; fALFF, fractional amplitude of low-frequency fluctuation; HC, healthy control; MNI, Montreal Institute of Neuroscience.

**TABLE 7:** Significant differences in ReHo between after acupuncture treatment BP patients and HC.

Brain regions	MNI coordinate			Voxel	*t*
	X	Y	Z		
Right precentral gyrus	33	–6	54	606	6.363
Right lingual gyrus	18	–51	–12	464	5.714
Right transverse temporal gyrus	48	–15	9	252	6.015
Left transverse temporal gyrus	–36	–30	15	168	5.071
Right thalamus	12	–21	3	74	5.146
Right middle frontal gyrus	39	42	33	1984	–3.389

BP, Bell’s palsy; HC, healthy control; MNI, Montreal Institute of Neuroscience; ReHo, regional homogeneity.

**FIGURE 6:**
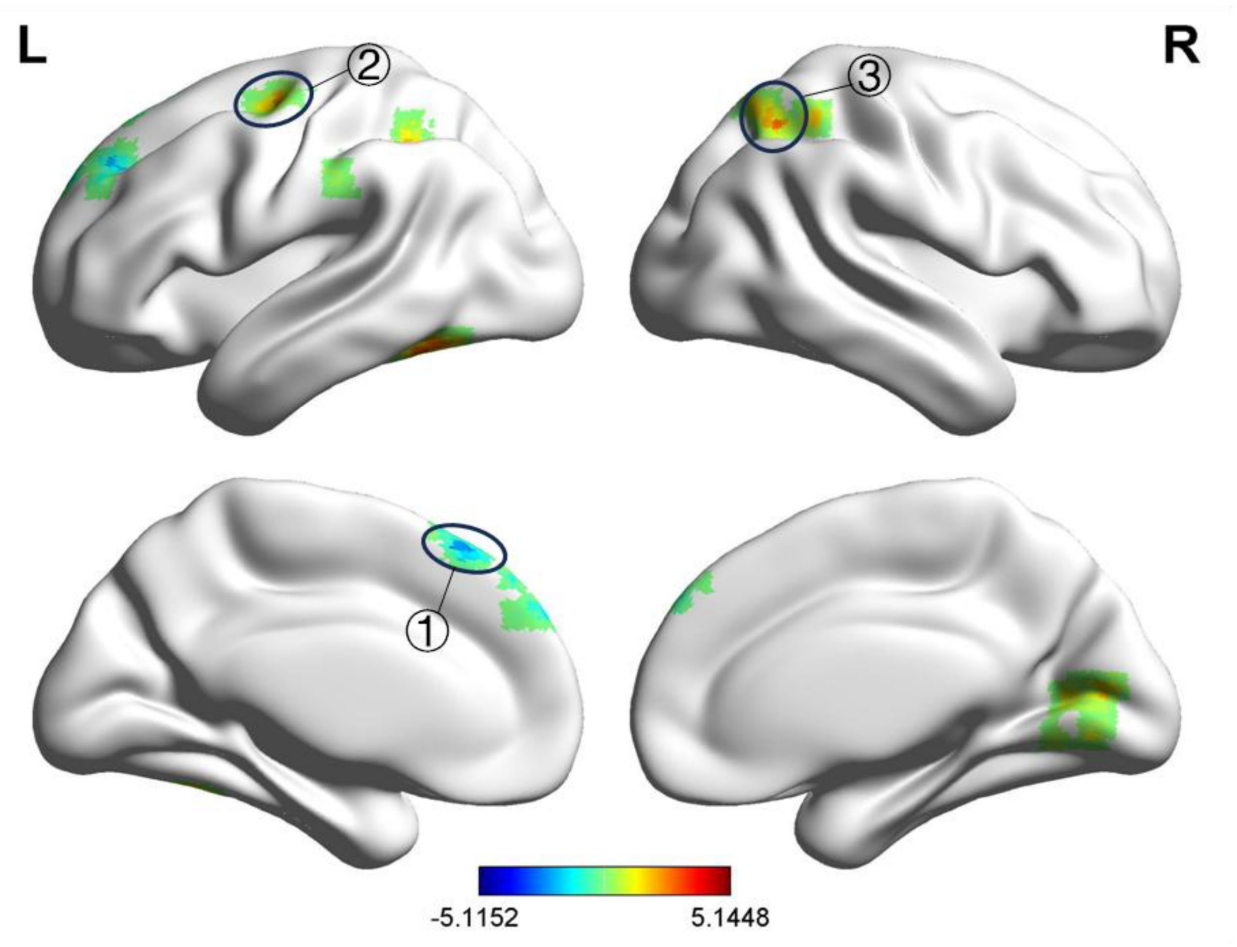
Significant differences in fALFF between after and before acupuncture treatment BP patients. The result was corrected for GRF with voxel *P* < 0.001, cluster *P* < 0.05. ① Left medial superior frontal gyrus, ② Left precentral gyrus, ③ Right angular gyrus.

**FIGURE 7:**
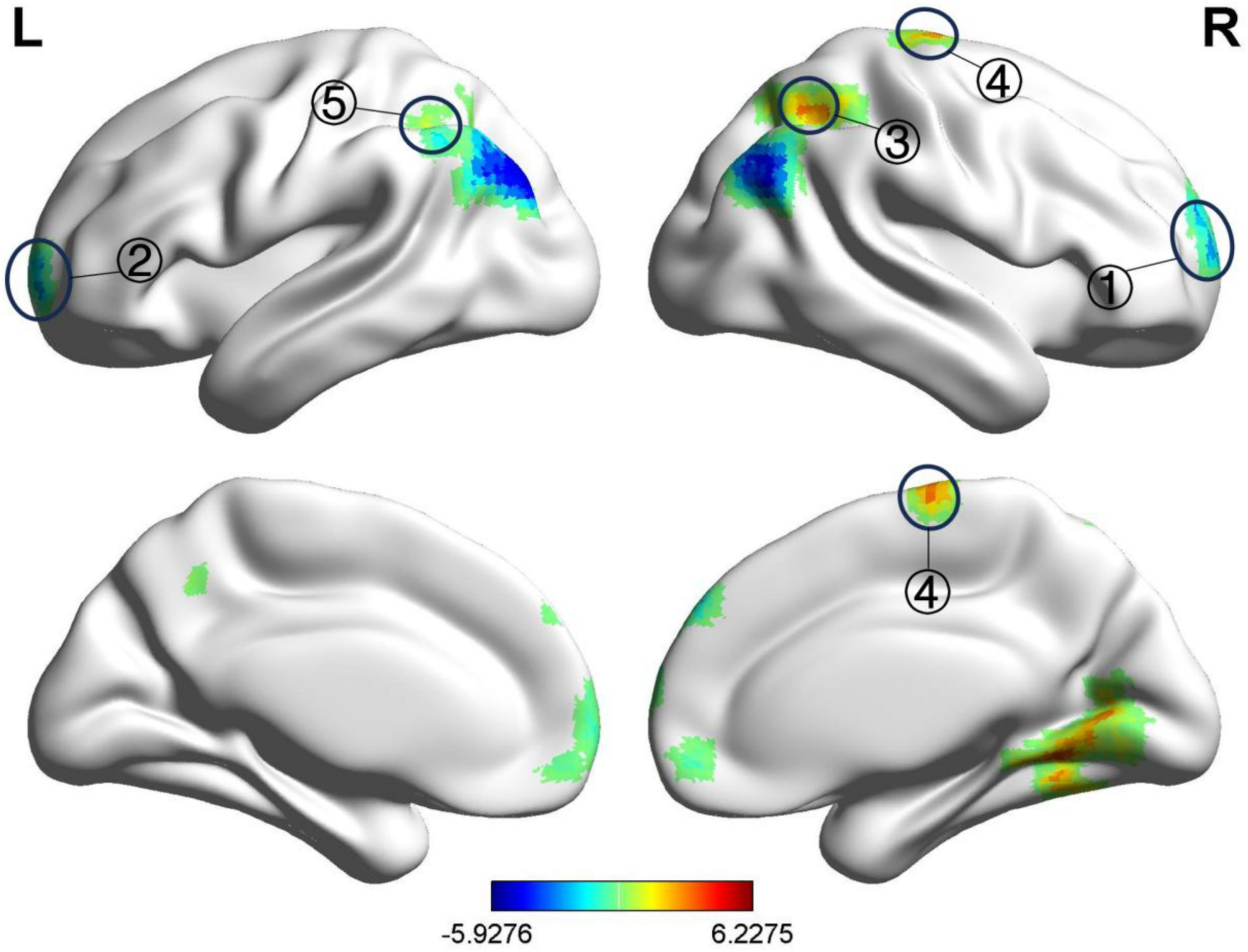
Significant differences in ReHo between after and before acupuncture treatment BP patients. The result was corrected for GRF with voxel *P* < 0.001, cluster *P* < 0.05. ① Right medial superior frontal gyrus, ② Left medial superior frontal gyrus, ③ Right inferior parietal lobule (angular gyrus), ④ Right precentral gyrus, ⑤ Left superior parietal gyrus.

Compared with before treatment, the fALFF value of the left medial superior frontal gyrus in the BP group decreased after treatment, while the fALFF value of the left precentral gyrus and the right angular gyrus increased, as shown in Table 8 and Figure 6. The ReHo values of the right and left medial superior frontal gyrus decreased, whereas the ReHo values of the right inferior parietal angular gyrus, right precentral gyrus, and left superior parietal gyrus increased, as shown in Table 9 and Figure 7. GRF correction was applied, with voxel-level *P* < 0.001 and cluster-level *P* < 0.01.

**TABLE 8:** Significant differences in fALFF between after and before acupuncture treatment BP patients.

Brain regions	MNI coordinate			Voxel	*t*
	X	Y	Z		
Left medial superior frontal gyrus	–3	54	45	43	–3.266
Left precentral gyrus	–30	–3	57	32	4.084

BP, Bell’s palsy; fALFF, fractional amplitude of low-frequency fluctuation; MNI, Montreal Institute of Neuroscience.

**TABLE 9:** Significant differences in ReHo between after and before acupuncture treatment BP patients.

Brain regions	MNI coordinate			Voxel	*t*
	X	Y	Z		
Right medial superior frontal gyrus	6	50	39	136	–3.480
Left medial superior frontal gyrus	–3	63	9	121	–3.480
Right inferior parietal angular gyrus	30	–45	51	74	6.228
Right precentral gyrus	15	–24	69	61	4.442
Left superior parietal gyrus	–18	–57	48	52	4.924

BP, Bell’s palsy; MNI, Montreal Institute of Neuroscience; ReHo, regional homogeneity.

## 4 Discussion

Using resting-state fMRI combined with two indicators, including fALFF and ReHo, this study systematically explored the clinical efficacy and the underlying central regulatory mechanism of acupuncture in the treatment of acute BP. Our analyses revealed that acupuncture treatment resulted in improved H-B scale grade, Sunnybrook scale score, and FDI score in BP patients, with an overall treatment effective rate of 96.4%. This suggests that acupuncture can significantly improve the patients’ clinical symptoms and quality of life, achieving remarkable efficacy in the treatment of BP. Moreover, our fMRI analyses indicated that there were significant differences in brain functional activities between BP patients and healthy individuals and that acupuncture treatment may improve abnormal brain functional activities by promoting functional reorganization of relevant brain regions.

Fractional amplitude of low-frequency fluctuation measures localized, spontaneous neuronal activity and can significantly inhibit non-specific signaling components in resting-state MRI, increasing sensitivity to local spontaneous brain activity (Wang Y. et al., 2021). ReHo measures the uniformity of brain functional regions under specific conditions, which can reflect the synchronization of spontaneous neural activity in brain regions, and higher ReHo values indicated higher consistency and centrality of the activity cadence of adjacent voxels within brain regions (Lv et al., 2018; Zang et al., 2004).

In the present study, the fALFF and ReHo values in the central posterior gyrus, the fALFF values in the middle frontal gyrus, the ReHo values in the left central anterior gyrus, and the ReHo values in the middle occipital gyrus increased with acupuncture treatment whereas the fALFF values in the right posterior cingulate gyrus decreased with acupuncture treatment, compared with the pretreatment values. The posterior central gyrus (Smith et al., 2009) is an important part of the cerebral cortex and is the main somatosensory area of the brain, which is also responsible for spatial orientation of sensations in various parts of the body. Changes in the central posterior gyrus in BP patients mean that this area not only has an increased level of spontaneous activity but also that these activities exhibit higher local synchrony. This change may reflect the brain’s adaptive response to sensory and motor impairment caused by facial paralysis, or it may be that the brain is trying to compensate for the loss of sensory and motor control caused by facial paralysis; activity and synchronicity in specific brain regions are increased to adjust and improve facial nerve dysfunction, with the observed changes reflecting the adaptive ability of the central nervous system. This finding may also indicate the underlying neuroplasticity, i.e., the ability to adjust brain structures and function, as a coping mechanism in BP. Whereas the frontal lobe (Sohn et al., 2004) is the cortical center of upper facial movement, the middle frontal gyrus is the core component of the premotor area, participating in motor planning and executive function (Niendam et al., 2012), and the anterior central gyrus is the primary motor cortex, which directly regulates somatic movement (Grefkes et al., 2008). The increased fALFF values found in the middle frontal gyrus and the increased ReHo values found in the anterior central gyrus indicate that the middle frontal gyrus strengthens the accuracy of facial movement planning by enhancing spontaneous activity, while the anterior central gyrus optimizes the efficiency of movement execution by improving internal synchronization; the two regions are linked to maintain the basic motor functions of the face to the greatest extent possible. The middle occipital gyrus is an important part of the optic cortex, involved in facial expression recognition and visual information processing (Fang et al., 2007; Gong et al., 2015; Kosslyn and Ochsner, 1994; Zhang et al., 2018), whereas the posterior cingulate gyrus is involved in self-awareness, emotional regulation, and sensory integration (Smith et al., 2009). Changes in the middle occipital gyrus may reflect the brain’s adaptive or compensatory mechanisms in pathological states, possibly related to changes in visual processing, facial expression recognition, or other tasks related to the occipital lobe function. This change is likely the brain’s response to injury, trying to restore or optimize function by increasing functional synchronicity in certain areas. The weakening of activity in the posterior cingulate gyrus may be associated with psychologic states, such as anxiety and social avoidance, caused by changes in facial appearance in patients with BP.

The results of this study showed that, compared with the HCs, BP patients had higher fALFF and ReHo values in the anterior central and lingual gyri, higher fALFF values in the central posterior gyrus, lower fALFF values in the superior frontal gyrus, higher ReHo values in the temporal transverse gyrus and thalamus, and lower ReHo values in the right middle frontal gyrus after acupuncture treatment. These changes suggest that acupuncture may be involved in modulating neuronal activity in these brain regions, thereby alleviating symptoms of facial paralysis and facial sensory and motor dysfunction in BP patients. Studies have shown (Yang et al., 2012) that acupuncture treatment in patients with BP results in enhanced connectivity between the primary sensory cortex, primary motor cortex, superior frontal gyrus, and premotor area of the middle frontal gyrus in their brain regions. The possible mechanism is that due to the loss of facial muscle function, BP patients are unable to fully execute the neural instructions sent by the primary sensory cortex and primary motor cortex. Consequently, higher-level central premotor areas are required to play a role in motor coordination, thereby strengthening the linkage between the primary sensory cortex and the primary motor cortex. This study found that acupuncture enhances the activity of the precentral gyrus, postcentral gyrus, lingual gyrus, transverse temporal gyrus, and thalamus. These brain regions are mainly responsible for regulating somatic motor function, sensory function, visual information processing, emotional regulation, and other aspects. Using resting-state fMRI, Kong et al. (2015) observed that acupuncture at Hegu (LI4) and Jiache (ST6) acupoints in BP patients could activate brain regions such as the precentral gyrus, postcentral gyrus, and middle frontal gyrus, which correspond to the somatosensory cortex, motor cortex, and prefrontal cortex. Additionally, a study by Liu et al. (2014) also showed that acupuncture treatment in BP patients results in functional reorganization in the premotor cortex and supplementary motor area of the cerebral cortex. These findings are largely consistent with the results of this study. Taken together, we speculate that acupuncture can regulate brain functional plasticity and enhance the functional connectivity between relevant brain regions by activating areas such as the somatosensory cortex, motor cortex, and prefrontal cortex of the brain. As a key region for functions like emotional regulation (Wang et al., 2017), the superior frontal gyrus exhibits reduced activity, suggesting that acupuncture alleviates negative emotions, such as anxiety and inferiority, by improving the facial appearance of patients, thereby restoring emotion-related cortical regions from a state of overactivation to a stable level, consistent with the decreased FDIS scores in patients. The decreased ReHo values in the middle frontal gyrus indicate a reduction in the compensatory overactivation of the middle frontal gyrus in BP patients and a return of brain function to a normal level; therefore, this finding should be considered a potential imaging manifestation of therapeutic efficacy. The present study reveals that BP patients still experience functional abnormalities in multiple brain regions after acupuncture treatment, suggesting that even after clinical recovery, BP patients still exhibit partial brain functional reorganization. A study by Klingner et al. (2011) corroborates this finding.

In the present study, the fALFF values in the right angular gyrus, the ReHo values in the right inferior parietal lobule (angular gyrus) and the left superior parietal gyrus, and the fALFF and ReHo values in the precentral gyrus increased whereas the fALFF and ReHo values in the superior frontal gyrus decreased after acupuncture treatment compared with the pretreatment values in BP patients. The parietal lobe (Yang and Gang, 2015; Zhang et al., 2019)—including the postcentral, superior parietal, and angular gyri and the inferior parietal lobule—is a critical brain region that participates in motor planning and coordination by interacting with the motor cortex; this is crucial in relearning fine motor control of the face in BP patients. Facial paralysis can impair patients’ facial perception, and the parietal lobe can aid in adjusting and adapting to these changes. The increases in the values of indicators related to the parietal lobe observed after treatment suggest that acupuncture enhances the neural activity capacity and synchrony in this region, which may strengthen functional coordination within and across distinct brain regions. The superior frontal gyrus (Wang et al., 2017) is involved in emotional regulation. The decreased fALFF and ReHo values observed in this region after acupuncture treatment may be associated with the improvement of emotional function observed following acupuncture treatment in BP patients Reduced brain regional activity may reflect a stabilization of patients’ emotional state following acupuncture treatment. The precentral gyrus (Song et al., 2024) is responsible for motor control and sensory information integration, particularly the fine movements of the face and other body parts. The increased fALFF and ReHo values detected in this gyrus after treatment indicate that acupuncture enhances the neural activity and functional coordination in this region. In BP patients, the enhanced function of the precentral gyrus may promote the repair of damaged nerves or improve the efficiency of neural signal transmission, thereby facilitating the recovery of facial motor function. The increased ReHo and fALFF values may also reflect the reorganization or optimization of brain functional networks after acupuncture treatment. Such changes may contribute to improved regulation of facial motor control by the brain, demonstrating the plasticity and self-repair capacity of the brain.

However, this study did not include a sham acupuncture control group; therefore, interference of natural disease recovery and psychological factors cannot be completely ruled out. A further analysis combining the disease characteristics and data features is as follows: on the one hand, although BP demonstrates natural recovery, the high recovery rate (27/55) of patients within 28 days in this study and the strong correlation between brain function changes and the acupuncture intervention cycle cannot be fully explained by “spontaneous recovery.” Peitersen (2002) pointed out that most patients with natural recovery require several weeks to months, and approximately 10%–15% of may have residual sequelae. In contrast, most patients in this study recovered faster and more completely, suggesting that acupuncture may have accelerated the recovery process. On the other hand, although psychological factors may affect the scores of subjective scales, the physiological responses induced by “Deqi” (needling sensation) during acupuncture and the improvement of objective brain function data from fMRI all indicate specific physiological regulation, rather than mere psychological suggestion.

Although this study provides preliminary evidence for the central mechanism underlying acupuncture treatment in BP patients, several study limitations should be addressed. First, the present study did not include a sham acupuncture group as a control; therefore, the interference of the natural course of recovery and the placebo effect cannot be completely ruled out. Although the impact of acupuncture treatment was evaluated by clinical and objective imaging indicators, future studies should include a sham acupuncture group as a control to further clarify the contributions of acupuncture’s “acupoint specificity” and the “*Deqi* effect.” Second, the study included a small sample size of 55 BP patients and 48 HCs and was conducted in a single center, introducing a potential selection bias. Future investigations should include multi-center studies with large cohorts and should consider relaxing the inclusion criteria to improve the generalizability of our findings. Third, the patients were followed for up to 28 days after treatment, with no long-term follow-up evaluations conducted every 3–6 months; therefore, we could not evaluate the persistence of brain functional changes or the stability of the clinical efficacy of acupuncture treatment. Furthermore, correlation analyses exploring associations between the indicators of brain function and clinical scores were not conducted, hindering our ability to identify specific brain regional changes that are directly associated with the observed therapeutic effects. Subsequent studies should conduct longitudinal fMRI follow-up evaluations in combination with correlation analysis to screen imaging biomarkers for the prediction of therapeutic efficacy. Finally, the study only used resting-state fMRI and did not include other techniques, such as diffusion tensor imaging or task-state fMRI. Therefore, we were not able to fully reveal the regulatory effect of acupuncture on brain structure–function networks. Future studies should employ multimodal imaging to analyze the mechanism of acupuncture in multiple dimensions.

In conclusion, this study provides preliminary evidence that acupuncture regulates brain function in BP patients. Changes in brain function emphasize that acupuncture treatment may exert a positive effect in BP patients by promoting better coordination between brain regions and enhancing the intensity of regional activity.

## 5 Conclusion

Significant differences were observed in brain functional activity, particularly in motor and sensory functions, between BP patients and healthy individuals. Acupuncture treatment may improve abnormal brain function activity while alleviating patients’ clinical symptoms by facilitating functional reorganization of the sensory cortex, motor cortex, and emotion-related brain regions.

## Data Availability

The original contributions presented in this study are included in this article/supplementary material, further inquiries can be directed to the corresponding author.
